# A public data set of spatio-temporal match events in soccer competitions

**DOI:** 10.1038/s41597-019-0247-7

**Published:** 2019-10-28

**Authors:** Luca Pappalardo, Paolo Cintia, Alessio Rossi, Emanuele Massucco, Paolo Ferragina, Dino Pedreschi, Fosca Giannotti

**Affiliations:** 1 ISTI-CNR, Pisa, Italy; 2 Department of Computer Science, University of Pisa, Pisa, Italy; 3 Wyscout, Chiavari, Italy

**Keywords:** Information technology, Medical research, Interdisciplinary studies

## Abstract

Soccer analytics is attracting increasing interest in academia and industry, thanks to the availability of sensing technologies that provide high-fidelity data streams for every match. Unfortunately, these detailed data are owned by specialized companies and hence are rarely publicly available for scientific research. To fill this gap, this paper describes the largest open collection of soccer-logs ever released, containing all the spatio-temporal events (passes, shots, fouls, etc.) that occured during each match for an entire season of seven prominent soccer competitions. Each match event contains information about its position, time, outcome, player and characteristics. The nature of team sports like soccer, halfway between the abstraction of a game and the reality of complex social systems, combined with the unique size and composition of this dataset, provide an ideal ground for tackling a wide range of data science problems, including the measurement and evaluation of performance, both at individual and at collective level, and the determinants of success and failure.

## Abstract (summary)

**Table:** 

Measurement(s)	physical activity
Technology Type(s)	visual observation method
Sample Characteristic - Location	Germany • Kingdom of Spain • French Republic • Italy • England

Machine-accessible metadata file describing the reported data: 10.6084/m9.figshare.9711164

## Background & Summary

Soccer analytics has attracted interest for a long time^1,2^. In the early 1950s Charles Reep collected statistics *by hand* to suggest that “the key to scoring goals is to transfer the ball as quickly as possible from back to front”^3^, thereby indirectly starting the long-ball movement in English football^4^.

Apart from a few sporadic attempts, it is only in recent years that soccer statistics have developed, thanks to sensing technologies that provide high-fidelity data streams extracted from every match. There are three main data sources available^5^: (i) *soccer-logs* describe the events that occur during a match and are collected through proprietary tagging software^6–9^; (ii) *video-tracking data* describe the trajectories of players during a match and are collected through video recordings^10,11^; (iii) *GPS data* describe the trajectories of players during training sessions and are collected through GPS devices worn by the players^12^. Despite this wealth of data, we cannot avoid noticing that soccer datasets are rarely available for scientific research. This limits the development of scientific methods for soccer analytics.

In this paper, we describe an open collection of soccer-logs that cover seven prominent male soccer competitions. The collection has been used recently during the Soccer Data Challenge initiative (https://sobigdata-soccerchallenge.it/) and, to the best of our knowledge, it is the largest collection of soccer-logs ever released to the public. Soccer-logs describe match *events*, each containing information about its type (pass, shot, foul, tackle, etc.), a time-stamp, the player(s), the position on the field and additional information (e.g., pass accuracy). We believe that these data are greatly beneficial to the scientific community because they can contribute to research in several directions, such as the ones we outline below.

### Performance analysis

Soccer-logs can be used to design algorithms for relevant problems such as the evaluation of performance and the discovery of tactics^1,5^. The problem of performance evaluation^9,13,14^ is crucial for many actors in the sports industry: from broadcasters who want to solicit critical analysis among the fans, to managers who want to monitor the quality of their players and scouts who aim to improve the retrieval of talents. The automatic discovery of tactics^6,15^ is also becoming a crucial task: while most tactical analyses are currently performed by reviewing video and matches in person, soccer-logs can be used to perform automatic discovery of tactics, simplifying the complex process of match analysis. While different approaches have been proposed in the literature using different datasets to attack these problems, our dataset is much larger and can serve as a common ground to compare and validate different solutions.

### Complex systems analysis

Two soccer teams in a match represent a complex system whose global behavior depends in subtle ways on the dynamics of the interactions among the players. Soccer-logs enable the representation of a team as a *network*, in which nodes represent players and the edges interactions between nodes, usually passes^7,14^. While the structure of passing networks is proven to be linked to a team’s strength^7,14^, the potential of a multiplex and dynamic representation of networks in soccer has not been much investigated^16^. Soccer-logs allow the definition of different types of interactions between both teammates and opponents by relying on the several event types they encode. Such a richness of information, combined with the dichotomous nature of soccer matches (where collaboration and competition coexist), provides an unprecedented opportunity to investigate novel aspects about the dynamics of complex networks.

### Science of success

The availability of a large dataset of sports performance also creates the opportunity to explore the relationship between performance and success, where a team’s success can be intended as its outcome in a competition and the player’s as their popularity or market value. While this relationship has been investigated for individual sports^17,18^, apart from a few attempts^19,20^ there is not much work for soccer, partly due to the absence of publicly available datasets of performance. Our dataset gives the unprecedented opportunity to answer fascinating questions like: ‘What are the tactical patterns of successful teams?’, ‘What are the factors influencing a player’s popularity and market value?’ and ‘To what extent is success predictable from the observable performance?’

## Methods

The data described in this paper have been collected and provided by Wyscout, a leading company in the soccer industry which connects soccer professionals worldwide, supports more than 50 soccer associations and more than 1,000 professional clubs around the world. The procedure of data collection is performed by expert video analysts (the operators), who are trained and focused on data collection for soccer, through a proprietary software (the tagger). The tagger has been developed and improved over several years and it is constantly updated to always guarantee better and better performance at the highest standards. Based on the tagger and the videos of soccer games, to guarantee the accuracy of data collection, the tagging of events in a match is performed by three operators, one operator per team and one operator acting as responsible supervisor of the output of the whole match. Optionally for near-live data delivery a team of four operators is used, one of them acting to speed up the collection of complex events which need additional and specific attributes or a quick review.

The tagging of a match consists of three main steps.

### Step 1: setting formations

At the beginning of the match, an operator sets the teams’ starting formations, the positions of the players on the pitch and their jersey number. The formation of a team consists of the list of players in the starting lineup and the list of players on the bench.

### Step 2: event tagging

**Fig. 1:**
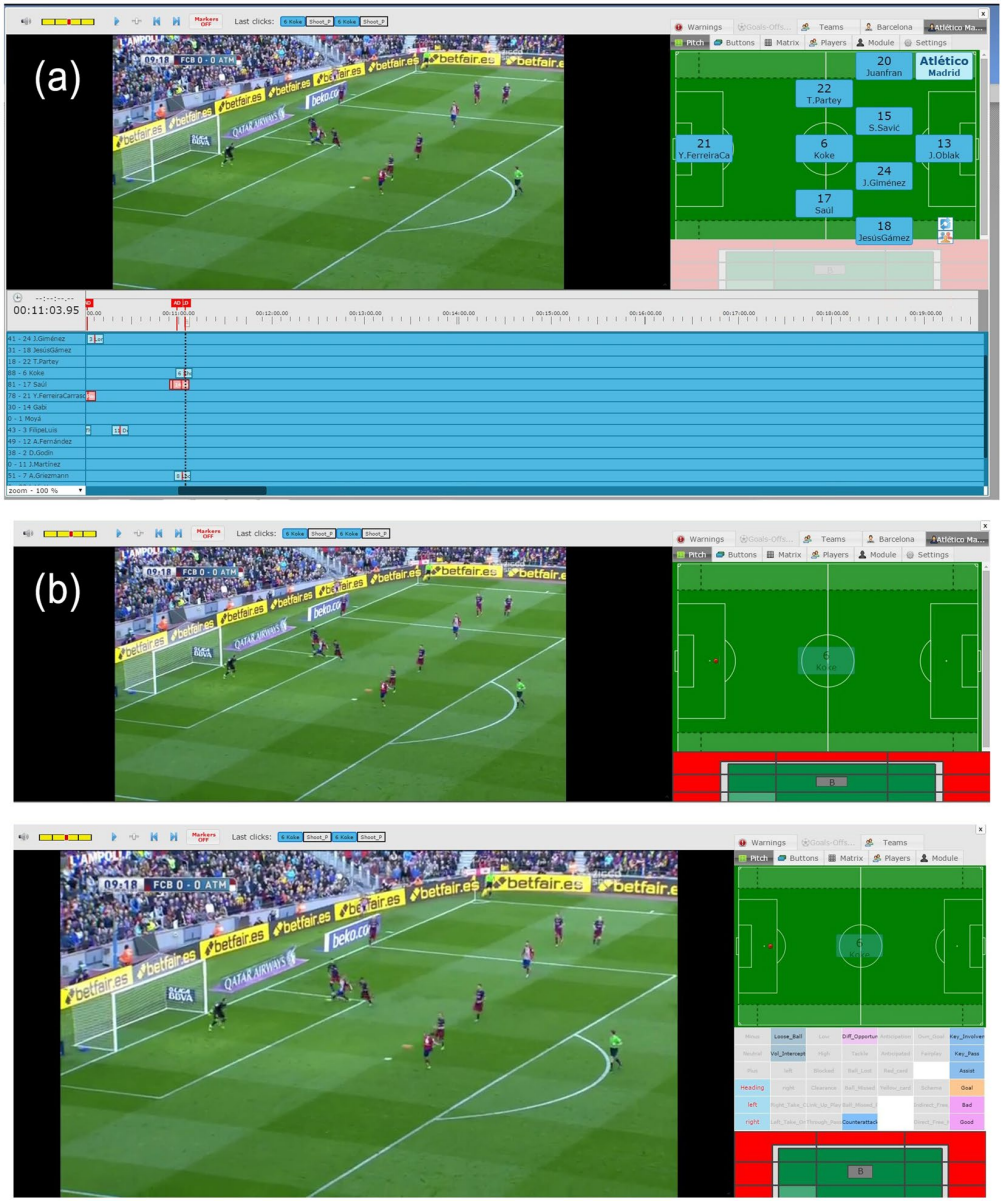
The process of tagging the soccer events from a match video. (**a**) Screenshot from the tagging software. An action is tagged by an operator via a special custom keyboard, thus creating a new event on the match timeline. (**b**) When the event position on the pitch is set, the shot specific input module appears (top). Event related input modules also appear for setting additional attributes of the occurring event (bottom).

For each ball touch in the match, the operator selects one player and creates a new event on the timeline. The operator then adds the type (e.g., pass, duel, shot, etc.) and subtype (e.g., a duel can be aerial or ground) of the event by using a special custom keyboard which gives operators the possibility to insert events and data in a streamlined way (Fig. 1a). The operator finally adds the coordinates on the pitch and all the additional attributes for the event. These can be different depending on the event type: such as pass high/low, foot, dribbling side and so forth (Fig. 1b). When a player shoots on goal, like in the example of Fig. 1b for player n.6 (Koke), the system asks the operator to fill a shot specific module that collects where the shot ends (on goal, out of goal, on post and exact position).

### Step 3: quality control

After the tagging, a procedure of quality control for each match is performed, mainly consisting of two different steps. The first step is automatic: an algorithm is used to avoid the majority of the errors made by operators, considerably reducing the margin of error. For example the algorithm matches the events tagged by both operators to crosscheck if they both collected events involving both teams, like duels, with the same positioning and interpretation. Similarly, the algorithm suggests events missed by the operators or searches for impossible combinations of event sequences. The second step of quality control is manual and supervised by quality controllers. It mainly consists of an in-depth check that is carried out once the match is completed. Going through each event of some sample matches, the controller can see and eventually correct any entered parameter. Sample matches for quality control are chosen by another algorithm in order to guarantee a well distributed and statistically meaningful coverage with respect to the kind and amount of analyzed matches.

## Data Records

The data sets are released under the CC BY 4.0 License and are publicly available on figshare^21^.

The data refer to season 2017/2018 of five national soccer competitions in Europe: Spanish first division, Italian first division, English first division, German first division, French first division. These competitions are the most important in Europe according to the UEFA country coefficient, which is used to rank the football associations of Europe and thus determine the number of clubs from an association that will participate in the UEFA Champions League and the UEFA Europa League (https://www.uefa.com/memberassociations/uefarankings/country/#/yr/2019). In addition, we provide the data of the World cup 2018 and the European cup 2016, which are competitions for national teams. In total, we provide seven data sets corresponding to information about all competitions, matches, teams, players, events, referees and coaches. Each data set is provided in JSON format (JavaScript Object Notation), an open-standard file format that uses human-readable and machine-processable text to transmit data objects consisting of attribute-value pairs and array data types (or any other serializable value). Table 1 shows the list of competitions we make available with their total number of matches, events and players. The data covers a total of around 1,941 matches, 3,251,294 events and 4,299 players.

**Table 1:** List of competitions with their total number of matches, events and players. We make available seven competitions, for a total of 1,941 matches, 3,251,294 events and 4,299 players.

Competition	#matches	#events	#players
Spanish first division	380	628,659	619
English first division	380	643,150	603
Italian first division	380	647,372	686
German first division	306	519,407	537
French first division	380	632,807	629
World cup 2018	64	101,759	736
European cup 2016	51	78,140	552
	1,941	3,251,294	4,299

**Table 2:** Event types, with their possible subtypes and tags.

type	subtype	tags
*pass*	cross, simple pass	accurate, not accurate, key pass, opportunity, assist, goal
*foul*		no card, yellow, red, 2nd yellow
*shot*		accurate, not accurate, block, opportunity, assist, goal
*duel*	air duel, dribbles, tackles, ground loose ball	accurate, not accurate
*free kick*	corner, shot, goal kick, throw in, penalty, simple kick	accurate, not accurate, key pass, opportunity, assist, goal
*offside touch*	acceleration, clearance, simple touch	counter attack, dangerous ball lost, missed ball, interception, opportunity, assist, goal

### Competitions

The competitions data set describes the seven competitions. Each competition is a document consisting of the following fields^21^ (see Wyscout documentation for further details at https://apidocs.wyscout.com/):

- **area**: denotes the geographic area associated with the league as a sub-document, using the ISO 3166-1 specification;
- **format**: the format of the competition. All competitions for clubs (Spanish first division, Italian first division, English first division, German first division, French first division) have value “Domestic league”. The competitions for national teams (World cup 2018, European cup 2016) have value “International cup”;
- **name**: the official name of the competition (e.g., Spanish first division, Italian first division, World cup 2018, etc.);
- **type**: the typology of the competition. It is “club” for the competitions for clubs (Spanish first division, Italian first division, English first division, German first division, French first division) and “international” for the competitions for national teams (World cup 2018, European cup 2016);
- **wyId**: the unique identifier of the competition, assigned by Wyscout.

Box 1 shows a document in the competitions data set referring to the Italian first division (“name”: “Italian first division”), a competition for clubs (“type”: “club” and “format”: “Domestic League”) held in Italy (see field “area”).

### Matches

The matches data set describes all the matches we make available. Each match is a document consisting of the following fields^21^ (see Wyscout documentation for further details at https://apidocs.wyscout.com/):

- **competitionId:** the identifier of the competition to which the match belongs to. It is a integer and refers to the field “wyId” of the competition document;
- **date** and **dateutc**: the former specifies date and time when the match starts in explicit format (e.g., May 20, 2018 at 8:45:00 PM GMT + 2), the latter contains the same information but in the compact format YYYY-MM-DD hh:mm:ss;
- **duration**: the duration of the match. It can be “Regular” (matches of regular duration of 90 minutes + stoppage time), “ExtraTime” (matches with supplementary times, as it may happen for matches in continental or international competitions), or “Penalities” (matches which end at penalty kicks, as it may happen for continental or international competitions);
- **gameweek**: the week of the league, starting from the beginning of the league;
- **label**: contains the name of the two clubs and the result of the match (e.g., “Lazio - Internazionale, 2–3”);
- **roundID**: indicates the match-day of the competition to which the match belongs to. During a competition for soccer clubs, each of the participating clubs plays against each of the other clubs twice, once at home and once away. The matches are organized in match-days: all the matches in match-day *i* are played before the matches in match-day *i* + 1, even tough some matches may be postponed to facilitate players and clubs participating in Continental or Intercontinental competitions. During a competition for national teams, the “roundID” indicates the stage of the competition (eliminatory round, round of 16, quarter finals, semifinals, final);
- **seasonId**: indicates the season of the match;
- **status**: it can be “Played” (the match has officially started and finished), “Cancelled” (the match has been canceled before it started), “Postponed” (the match has been postponed and no new date and time is available yet) or “Suspended” (the match has been suspended by the referee because of conditions which make it impossible to continue play, such as inclement weather or power failure, and no new date and time is available yet);
- **venue**: the stadium where the match was held (e.g., “Stadio Olimpico”);
- **winner**: the identifier of the team that won the game, or 0 if the match ended with a draw;
- **wyId**: the identifier of the match, assigned by Wyscout;
- **teamsData**: it contains several subfields describing information about each team that is playing that match, such as lineup, bench composition, list of substitutions, coach and scores:
  - **hasFormation**: it has value 0 if no formation (lineups and benches) is present, and 1 otherwise;
  - **score**: the number of goals scored by the team during the match (not counting penalties);
  - **scoreET**: the number of goals scored by the team during the match, including the extra time (not counting penalties);
  - **scoreHT**: the number of goals scored by the team during the first half of the match;
  - **scoreP**: the total number of goals scored by the team after the penalties;
  - **side**: the team side in the match (it can be “home” or “away”);
  - **teamId**: the identifier of the team;
  - **coachId**: the identifier of the team’s coach;
  - **bench**: the list of the team’s players that started the match on the bench and some basic statistics about their performance during the match (goals, own goals, cards);
  - **lineup**: the list of the team’s players in the starting lineup and some basic statistics about their performance during the match (goals, own goals, cards);
  - **substitutions**: the list of team’s substitutions during the match, describing the players involved and the minute of the substitution.

Box 2 shows a document describing a match between Lazio and Internazionale (“label”: “Lazio - Internazionale, 2–3”) of the Italian first division (“competitionId”: 524), held on May 20th 2018 (see fields “date” and “dateutc”). Box 3 shows the structure of the formation subdocument for one of the teams, which includes the list of players on the bench, the list of players in the starting lineup and the list of substitutions made by the team.

### Teams

The teams data set describes the clubs or national teams playing in the seven competitions. Each document in this data set consists of the following fields^21^ (see Wyscout documentation for further details at https://apidocs.wyscout.com/):

- **city**: the city where the team is located. For national teams it is the capital of the country;
- **name**: the common name of the team;
- **area**: information about the geographic area associated with the team;
- **wyId**: the identifier of the team, assigned by Wyscout;
- **officialName**: the official name of the team (e.g., Juventus FC);
- **type**: the type of the team. It is “club” for teams in the competitions for clubs (Spanish first division, Italian first division, English first division, German first division, French first division.) and “national” for the teams in international competitions (World cup 2018, European cup 2016);

Box 4 shows a document describing team Juventus (“name”: “Juventus”) which is located in Turin (“city”: “Torino”) in Italy (see field “area”).

### Players

The players data set describes all players in the seven competitions^21^ (see Wyscout documentation for further details at https://apidocs.wyscout.com/). Each document in this data set consists of the following fields:

- **birthArea**: geographic information about the player’s birth area;
- **birthDate**: the birth date of the player, in the format “YYYY-MM-DD”;
- **currentNationalTeamId**: the identifier of the national team where the players currently plays;
- **currentTeamId**: the identifier of the team the player plays for. The identifier refers to the field “wyId” in a team document;
- **firstName**: the first name of the player;
- **lastName**: the last name of the player;
- **foot**: the preferred foot of the player;
- **height**: the height of the player (in centimeters);
- **middleName**: the middle name (if any) of the player;
- **passportArea**: the geographic area associated with the player’s current passport;
- **role**: the main role of the player. It is a subdocument containing the role’s name and two abbreviations of it;
- **shortName2**: the short name of the player;
- **weight**: the weight of the player (in kilograms);
- **wyId**: the identifier of the player, assigned by Wyscout.

Box 5 shows a document describing player Lionel Andres Messi Cuccittini (“shortName2”: “L. Messi”), who was born in Argentina (see field “birthArea”) and has the Spanish passport (see “passportArea”). From the document we observe that Messi’s preferred foot is the left foot (“foot”: “left”), his height and weight are 170 centimeters (“height”: 170) and 72 kilograms (“weight”: 72) respectively, he preferably plays as a forward (see field “role”) and he was born in 1987 (“birthDate”: “1987-06-24”).

### Events

The events data set describes all the events that occur during each match^21^ (see Wyscout documentation for further details at https://apidocs.wyscout.com/). Each event document contains the following information:

- **eventId**: the identifier of the event’s type. Each eventId is associated with an event name (see next point);
- **eventName**: the name of the event’s type. There are seven types of events (see Table 2): pass, foul, shot, duel, free kick, offside and touch;
- **subEventId**: the identifier of the subevent’s type. Each subEventId is associated with a subevent name (see next point);
- **subEventName**: the name of the subevent’s type. Each event type is associated with a different set of subevent types (see Table 2);
- **tags**: a list of event tags, each describing additional information about the event (e.g., accurate). Each event type is associated with a different set of tags (see Table 2). The Wyscout documentation provides a mapping of the tag identifiers to the corresponding names and descriptions (https://apidocs.wyscout.com/);
- **eventSec**: the time when the event occurs (in seconds since the beginning of the current half of the match);
- **id**: a unique identifier of the event;
- **matchId**: the identifier of the match the event refers to. The identifier refers to the field “wyId” in a match document;
- **matchPeriod**: the period of the match. It can be “1H” (first half of the match), “2H” (second half of the match), “E1” (first extra time), “E2” (second extra time) or “P” (penalties time);
- **playerId**: the identifier of the player who generated the event. The identifier refers to the field “wyId” in a player document;
- **positions**: the origin and destination positions associated with the event. Each position is a pair of coordinates (*x*, *y*). The *x* and *y* coordinates are always in the range [0, 100] and indicate the percentage of the field from the perspective of the attacking team. In particular, the value of the *x* coordinate indicates the event’s nearness (in percentage) to the opponent’s goal, while the value of the *y* coordinates indicates the event’s nearness (in percentage) to the right side of the field;
- **teamId**: the identifier of the player’s team. The identifier refers to the field “wyId” in a team document.

Box 6 shows an example of pass event (“eventId”: 8, “eventName”: “Pass”) generated by player 3344 (“playerId”: 3344) of team 3161 (“teamId”: 3161) in match 2576335 (“matchId”: 2576335) at second 2.41 of the first half of the match (“eventSec”: 2.4175, “matchPeriod”: “1H”). The pass started at position (49, 50) of the field and ended at position (38, 58) of the field (see field “positions”). Moreover, the pass was accurate as indicated by the presence of tag 1801 (field “tags”).

### Coaches

The coaches data set describes all coaches of the clubs and the national teams of the seven competitions we make available^21^ (see Wyscout documentation for further details at https://apidocs.wyscout.com/). It consists of the following fields:

- **wyId**: the identifier of the coach, assigned by Wyscout.
- **shortName**: the short name of the coach;
- **firstName**: the first name of the coach;
- **middleName**: the middle name (if any) of the coach;
- **lastName**: the last name of the coach;
- **birthDate**: the birth date of the coach, in the format “YYYY-MM-DD”;
- **birthArea**: geographic information about the coach’s birth area;
- **passportArea**: the geographic area associated with the coach’s current passport;
- **currentTeamId**: the identifier of the coach’s team. The identifier refers to the field “wyId” in a team document.

Box 7 shows a document describing coach Maurizio Sarri (“shortName”: “M. Sarri”), who was born in Italy (see field “birthArea”), has the Italian passport (see “passportArea”) and he was born in 1959 (“birthDate”: “1959-01-10”).

### Referees

The referees data set describes all referees in the national and international competitions we make available^21^ (see Wyscout documentation for further details at https://apidocs.wyscout.com/). It consists of the following fields:

- **wyId**: the identifier of the referee, assigned by Wyscout.
- **shortName**: the short name of the referee;
- **firstName**: the first name of the referee;
- **middleName**: the middle name (if any) of the referee;
- **lastName**: the last name of the referee;
- **birthDate**: the birth date of the referee, in the format “YYYY-MM-DD”;
- **birthArea**: geographic information about the referee’s birth area;
- **passportArea**: the geographic area associated with the referee’s current passport;

Box 8 shows a document describing referee William Collum (“shortName”: “W. Collum”), who was born in Scotland (see field “birthArea”), has a Scottish passport (see “passportArea”) and was born in 1979 (“birthDate”: “1979-01-18”).

Box 1 Example of document in the competitions data set describing the Italian first division.

Box 2 Example of a document in the matches data set describing a match between Lazio and Internazionale.

Box 3 Example of team document describing the club Juventus FC.

Box 4 Information about a team in the teams data set.

Box 5 Information about a player contained into the players data set.

Box 6 Information about a pass event contained into the events data set.

Box 7 Information about a coach in the coaches data set.

Box 8 Information about a referee contained into the referee data set.

## Technical Validation

**Fig. 2:**
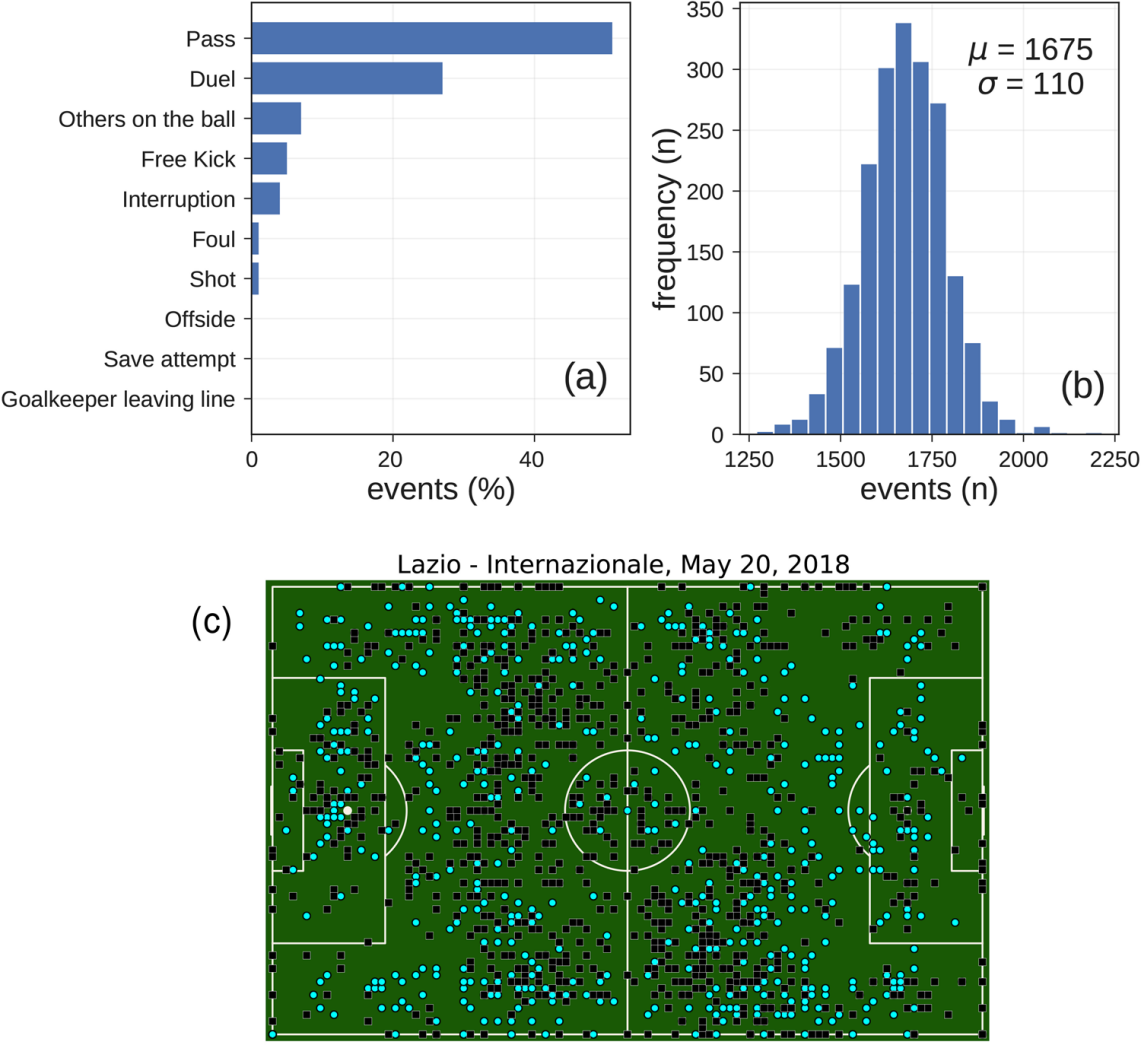
Statistics of the events data set. (**a**) Frequency of events per type. (**b**) Distribution of the number of events in soccer matches. (**c**) Events produced by the two teams in the match Lazio (cyan points) vs. Internazionale (black squares). The events are plotted on the position of the field where they occurred.

In general, based on the events data set, a soccer match consists of an average of 1,682 ± 101 events (Fig. 2b), with an inter-time between two consecutive events of 3.59 ± 7.42 seconds. There are on average 59 ± 29 events observed for a player in a match, one every 78.78 ± 105.64 seconds, confirming that soccer players are typically in ball possession for less than two minutes^22^. Passes are the most frequent events, accounting for around 50% of the total events (Fig. 2a). Duels (e.g., tackles and dribbles) are the second most frequent events (≈28%), while shots account for about 1.5% of the total events. The goals scored, the most important events in soccer since they determine a match outcome, are the rarest ones accounting for less than 1% of the total number of events. We provide an example of all the events (1,620) observed for the match “Lazio - Internazionale” of the Italian first division (May 20, 2018), plotted on the position of the field where they have occurred (Fig. 2c).

### Spatial dimension

**Fig. 3:**
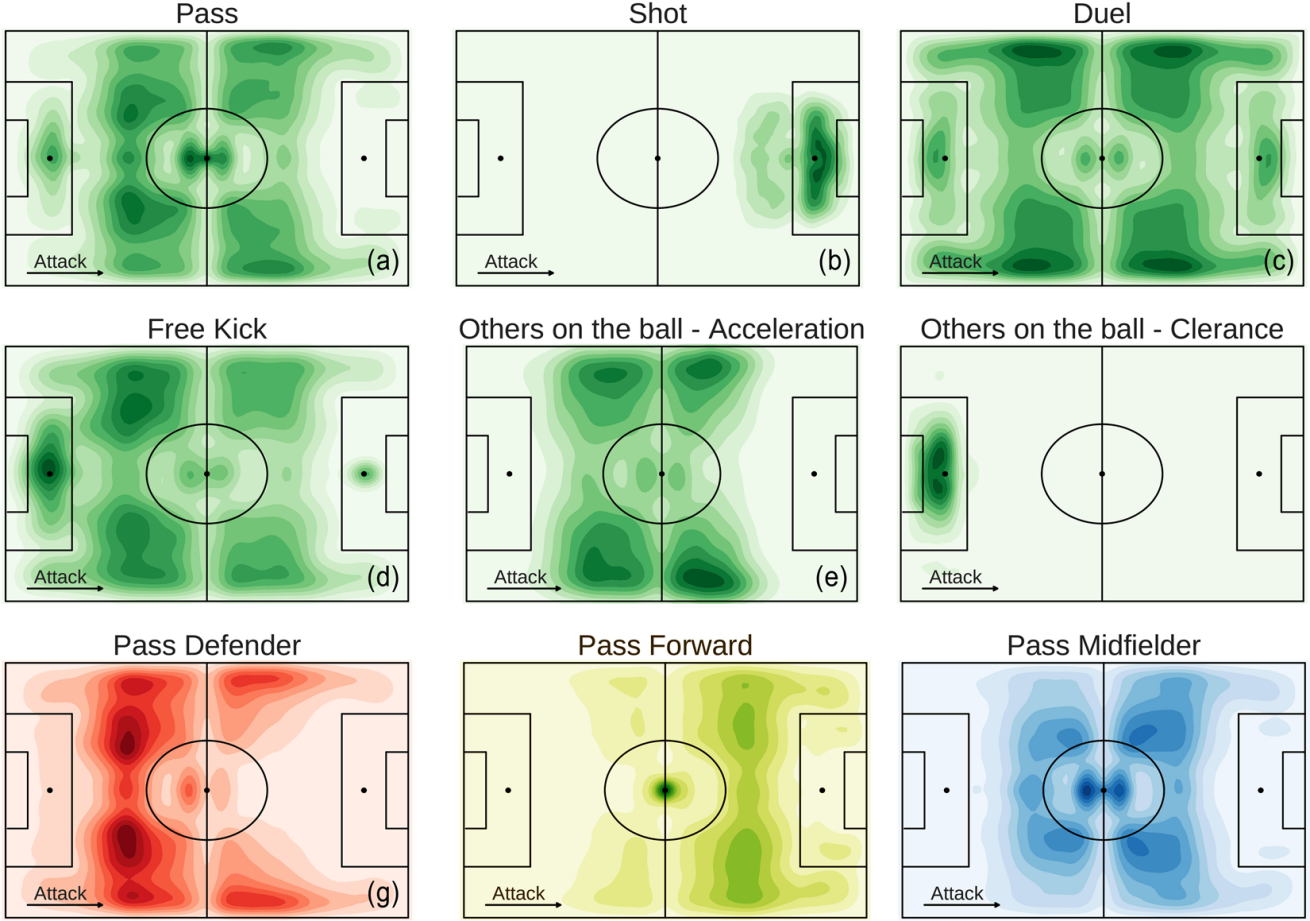
Distribution of positions per event type. (**a–f**) Kernel density plots showing the distribution of the events’ positions during match. The darker is the green, the higher is the number of events in a specific field zone. (**g–i**) Distribution of the passes’ position during a match for each player’s role. The darker is the color, the higher is the number of passes in a specific field zone.

By looking at the position of the field *where* the events occur, we can investigate interesting aspects of a soccer match, such as the spatial distribution of players and events. For example the kernel density plot in Fig. 3a shows that passes are distributed mostly in the center of the field, where actually most of the match takes place. As one could expect, we observe differences in the spatial distribution of events when we select the players by their role: while the events of forwards are observed mainly in the opponent’s half of the field (Fig. 3h), the events of defenders are observed mostly in the own half and on the sides of the field (Fig. 3g). Similarly, as expected the spatial distribution of events change with their type: attacking events (e.g., shots) are mostly observed close to the opponent’s goal (Fig. 3b), while defensive events (e.g., clearances) are mostly observed close to the team’s own goal (Fig. 3f). The spatial dimension of match events can provide us with information about a player’s behavior during a match, giving for example the possibility to determine a player’s profile from his average position during a match^13^.

### Temporal dimension

**Fig. 4:**
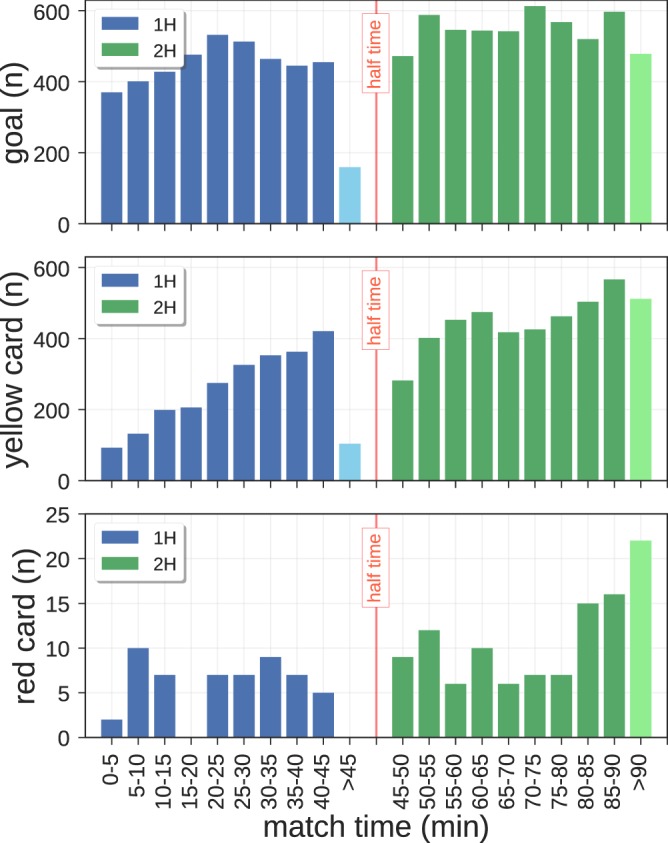
In-match evolution of the number of events. Number of events (i.e., goals on the top plot, yellow cards in the middle plot and the red cards in the bottom plot) that occur in all the matches in the data set, with time windows of 5 minutes.

By looking at *when* the events occur during a game, we can investigate interesting dynamics of teams and players. For example, Fig. 4 shows that goals are scored more frequently in the second half of the match^23,24^, mirroring several of the possible factors that could affect scoring, such as a decrease of attention by the defenders towards the end of the match due to a loss of stamina, or a more offensive attitude of the opponents who try to win or equalize the match. Similarly, we observe that the frequency of other rare events like yellow and red cards is the highest in the recovery time. This aspect could highlight the presence of a bias by the referees who are less prone to award a card in the beginning of a match (as suggested in^25^), a reduction of stamina or an increment of aggression of players at the end of the match.

**Fig. 5:**
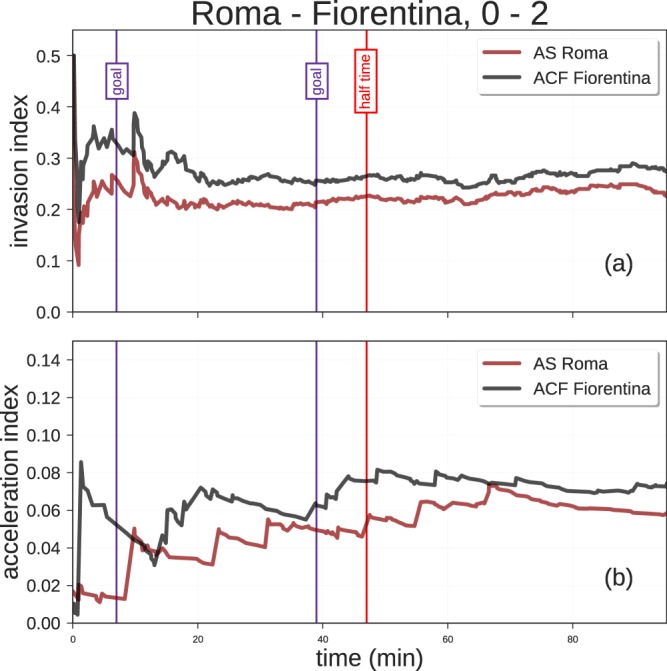
Invasion index and acceleration index for a game in the match data set. Bold lines represent the rolling mean of, respectively, invasion index (**a**) and acceleration index (**b**), while thin lines represent the individual values computed for each possession phase of each team. Purple vertical lines refer to the two goal scored by Fiorentina during the match, while the red vertical line indicates the half time of the match.

Another aspect that can be investigated by combining the spatial and the temporal dimensions of soccer-logs are the so-called *invasion index*, a measure of how close to the opponent’s goal a team plays during a match (i.e., its dangerousness), and *acceleration index*, a measure of how fast a team reaches the closest position to the opponent’s goal^26^. By exploiting the spatial and temporal dimension of soccer-logs, the invasion index can be computed on each possession phase, which is defined as a sequence of events on the ball made by a team before the opponents gain the possession. To compute the invasion index of a possession phase we compute: (i) for each event in the possession phase, the probability of scoring from the position where the event occurs (defined as the fraction of goals that have been scored from that position); (ii) we take the highest of these probabilities. A team’s overall invasion index during a match is simply the average invasion index across its possession phases. Figure 5 shows the invasion and acceleration index of the teams throughout the match Roma - Fiorentina (0–2), played on April 7, 2018. We observe that Fiorentina has on average a higher invasion index than Roma (0.27 ± 0.33 and 0.23 ± 0.31, respectively).

A team’s average acceleration index is another measure of its playing efficacy during a match. The acceleration index of a team’s possession phase is computed as the ratio between its invasion index and the square of the time between the first event and most dangerous event of the possession phase. A team’s average acceleration index during a match is the average acceleration index across its possession phases. Similarly to the invasion index, Fiorentina has a higher average acceleration than Roma (Roma: 0.06 ± 0.16, Fiorentina: 0.07 ± 0.15).

Both the invasion and the acceleration indices show that Fiorentina (the winner of the match) was more dangerous during the match, staying closer to the opponent’s goal and reaching dangerous zones faster than Roma.

### Team analysis

**Fig. 6:**
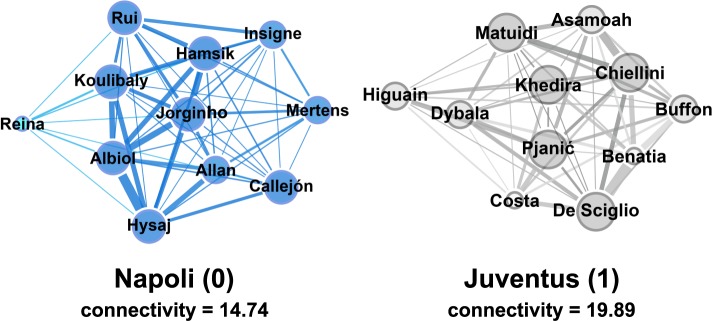
Representation of the player passing networks of the match Napoli-Juventus. Nodes represent players, edges represent passes between players. The size of the nodes reflects the number of ingoing and outgoing passes (i.e. node’s degree), while the size of the edges is proportional to the number of passes between the players.

Soccer-logs enable the analysis of the *interactions* between players through the reconstruction of a team’s *passing network*^7,14^, a representation of the movements of the ball between teammates during a match. A passing network allows identifying the key players in the team, i.e., the ones having more connections to the teammates or a high passing activity^27,28^. Figure 6 shows two examples of a team passing network for the match Napoli - Juventus (Italian first division). Although Napoli engaged in more passes than Juventus (666 vs. 332), the two passing networks show similar average weighted out-degrees (1.01 ± 0.93% and 1.10 ± 0.84%, respectively). However, Juventus’ playing style resulted in a higher *connectivity*^29^, defined as the network’s second smallest eigenvalue (i.e., a root of the characteristic equation of a matrix). This value indicates the robustness of a team, i.e., the strength of the links between its players. As a matter of fact, large values of connectivity between teammates are associated with a better overall team performance.

The reconstruction of passing networks from soccer-logs enables several performance analyses^7^. For example, by using the passing network and the players’ position during a pass it is possible to identify the most efficient tactical patterns across teams^30,31^.

### Player analysis

**Fig. 7:**
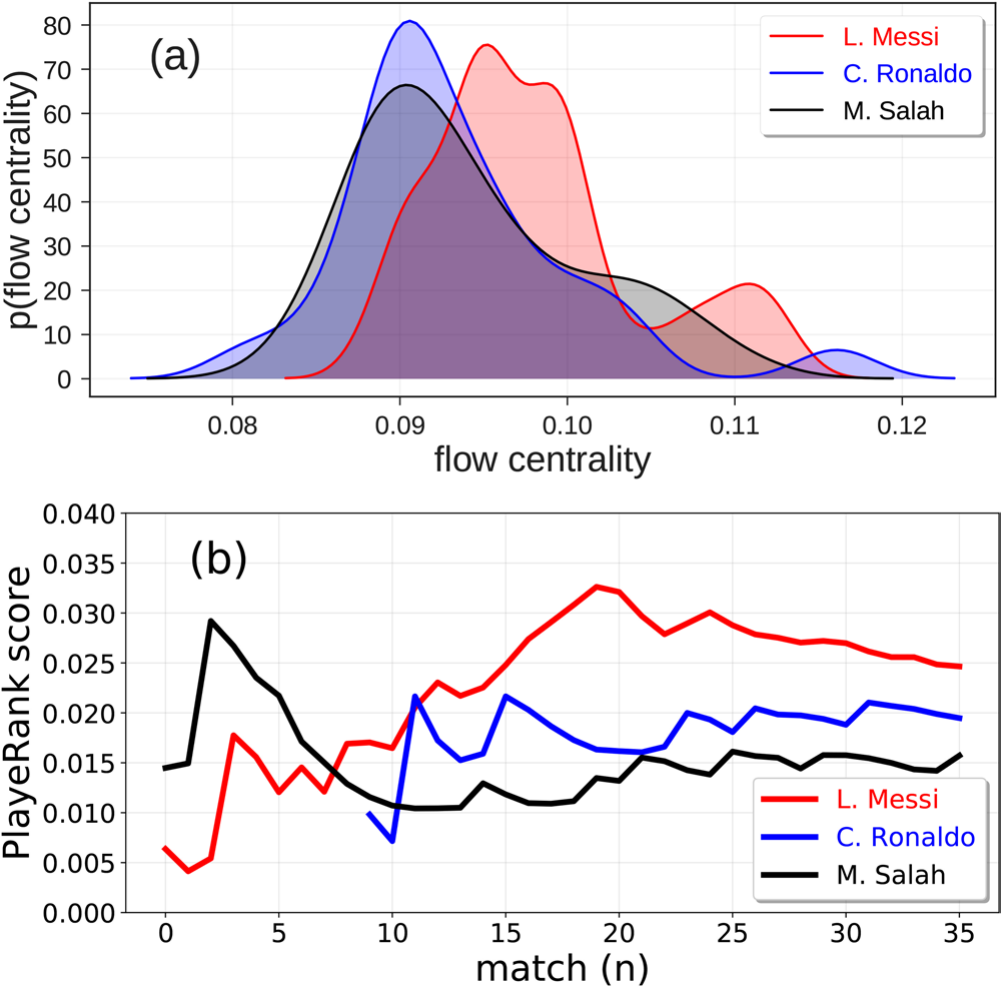
Distribution of flow centrality and PlayeRank score for three top players. (**a**) Distribution of the flow centrality of L. Messi (red line), C. Ronaldo (blue line) e M. Salah (black line) during the soccer season 2017/2018. (**b**) Performance quality calculated as the PlayeRank score of L. Messi (red line), C. Ronaldo (blue line), and M. Salah (black line).

Soccer-logs can be used to compare the *performance* of players and track their evolution in time. As an example, we compare three forwards with different characteristics – L. Messi (FC Barcelona), C. Ronaldo (Juventus FC) and M. Salah (Liverpool). We observe that L. Messi has the highest passing activity: while he produces 49 ± 19 passes per match on average, C. Ronaldo and M. Salah produce 26 ± 6 and 25 ± 9 passes per matches, respectively. Additionally, we observe that L. Messi engages in more duels per match (25 ± 8) than C. Ronaldo and M. Salah (15 ± 5 and 21 ± 7 duels per match). The data we release to the public also enable the computation of several performance metrics, such as Flow Centrality^14^ and PlayeRank^13^. A player’s flow centrality in a match is defined as his betweenness centrality in the passing network^14^. Figure 7a shows the distribution of flow centrality of L. Messi, C. Ronaldo and M. Salah for the matches in season 2017/2018. L. Messi results in a higher flow centrality (0.10 ± 0.01) than C. Ronaldo and M. Salah (0.09 ± 0.01 and 0.09 ± 0.01, respectively).

The performance quality of the players during the season can be assessed using PlayeRank, a data-driven framework that offers a principled multi-dimensional and role-aware evaluation of the soccer players’ performance quality in a match or in a series of matches^13^. Figure 7b shows that the three aforementioned players have different performance trends during the season. M. Salah obtained his best performance in the first part of the season, then decreasing during the course of the season. In contrast, L. Messi significantly increases his performance quality throughout the season while C. Ronaldo, who was not playing the first part of the season due to an injury, has on average a performance quality slightly higher than Salah but lower than Messi. We can conclude that, according to two measures computed on soccer-logs, Messi performs the best both in terms of passing centrality and performance quality.

## Data Availability

The code to reproduce the plots in the paper is available upon request by writing at luca.pappalardo@isti.cnr.it or info@sobigdata.eu.
